# Submerged and Impacted Primary Molars

**DOI:** 10.5005/jp-journals-10005-1080i

**Published:** 2010-09-15

**Authors:** SK Mishra, MK Jindal, Rajat Pratap Singh, Thomas R Stark, GS Hashmi

**Affiliations:** 1 Reader, Department of Conservative Dentistry and Endodontics, ZA Dental College, Aligarh Muslim University, Aligarh, Uttar Pradesh, India; 2 Reader and Chairman, Department of Pedodontics, ZA Dental College, Aligarh Muslim University, Aligarh, Uttar Pradesh, India; 3 Private Practice, The Gentle Dental Home, Ramghat Road, Aligarh, Uttar Pradesh, India; 4 Major, US Army, Weed Army Community Hospital, Fort Irwin, California, USA; 5 Senior Assistant Professor, Department of Oral and Maxillofacial Surgery, ZA Dental College, Aligarh Muslim University Aligarh, Uttar Pradesh, India

**Keywords:** Submerged, Deciduous, Ankylosed.

## Abstract

Submerged tooth is the one that is depressed below the occlusal plane. Dental ankylosis is thought to be a major cause of submergence. Submerged deciduous teeth have the potential to cause malocclusion not only by prevention of their exfoliation and subsequent replacement by permanent teeth but also by causing tilting of proximal teeth and extrusion of opposing tooth. The purpose of this report is to present three different cases of submerged deciduous teeth and their clinical effects.

## INTRODUCTION

Submerged deciduous teeth means the affected teeth do not come to the level of adjacent normal occluding teeth or submerged teeth are always 0.5 mm or more below the intact marginal ridges of the adjacent teeth.^1^ This incidence occurs after eruption/emergence of teeth in the oral cavity.

Trauma is the most common cause damaging either the dental follicle or the developing periodontal ligament but the exact etiology is unknown. If it happens then the eruption of the tooth ceases and it becomes ankylosed in the jaw bone. Because of continued eruption of the neighboring teeth and increase in the height of alveolar bone, the ankylosed tooth may be either “shortened” or submerged in the alveolar bone.

The prevalence of submerged deciduous teeth in children varies from 1.3 to 3.5%. The most commonly affected teeth are the deciduous mandibular second molars.^2-4^ This process prevents their exfoliation.

## CASE 1

A 9-year-old boy came to the dental OPD of ZA Dental College, AMU, Aligarh (UP), India with a complaint of cheek bite on his left side. On examination, the left mandibular deciduous second molar was absent and the permanent mandibular left 1st molar was mesially tilted along with supra-eruption of maxillary left deciduous second molar resulting in disturbed occlusion, responsible for cheek bite and ulceration.

**Fig. 1:**
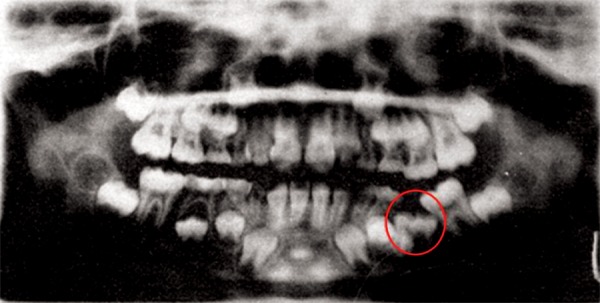
Panoramic radiograph showing a submerged deciduous mandibular left second molar

Panoramic radiograph demonstrated that deciduous second molar was submerged and there was severe tilting of permanent mandibular 1st molar (Fig. 1). Hence, early removal of the deciduous molar was planned in order to prevent the development of malocclusion in the future.

The tooth has to be removed carefully while saving the erupting permanent 2nd premolar.

## CASE 2

A 9-year-old boy came to the OPD of ZA Dental College, AMU, Aligarh with a complaint of carious upper tooth and missing lower posterior teeth.

On clinical examination, it was found that there was caries in the upper left deciduous second molar and the lower deciduous second molars along with the permanent first molars were absent. An OPG was advised.

**Fig. 2:**
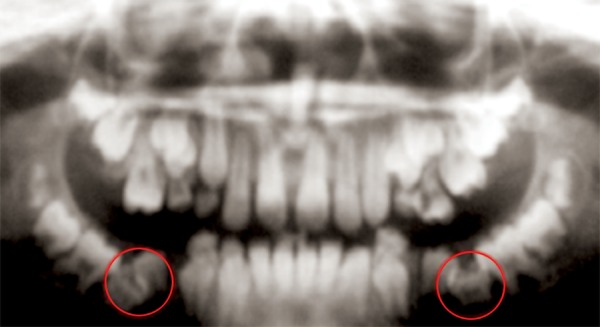
Panoramic radiograph showing bilateral submerged primary second molars

**Fig. 3:**
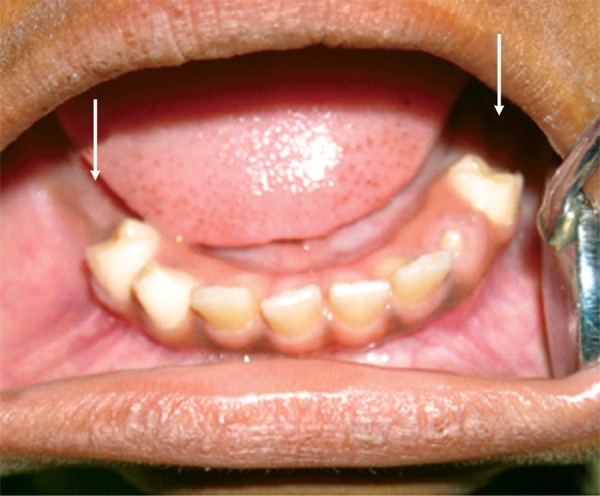
Intraoral image showing bilaterally absent mandibular primary second molars

The panoramic radiograph (Fig. 2) revealed that the lower deciduous second molars were completely submerged/ ankylosed in the bone and the permanent mandibular first molars were present showing signs of delayed eruption (Fig. 3).

## CASE 3

A 7-year-old healthy male, afraid of the dentist, came to the Weed Army Community Hospital, Fort Irwin (CA), USA complaining that “His tooth is sinking”.

**Fig. 4:**
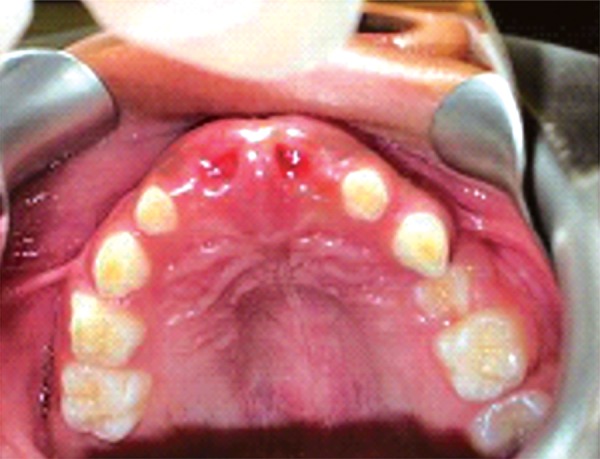
Intraoral image showing submerged mandibular primary left first molar

**Fig. 5:**
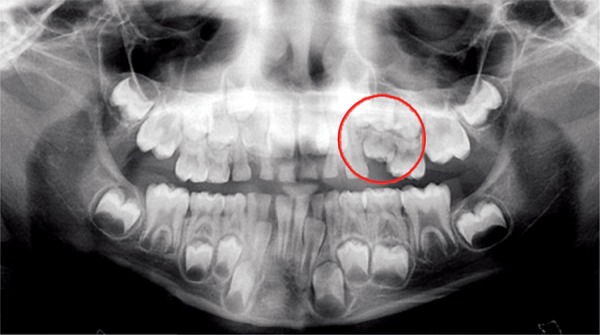
OPG showing ankylosed primary maxillary left first molar

**Fig. 6:**
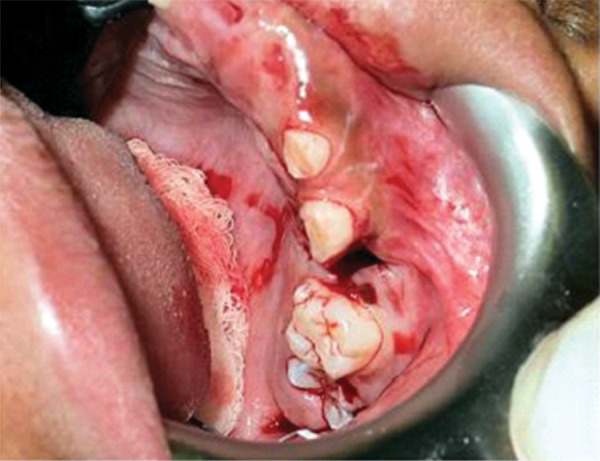
Ankylosed primary molar extracted under general anesthesia

**Fig. 7:**
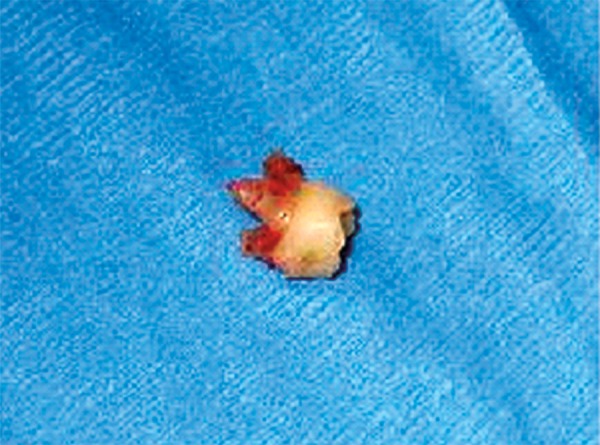
Extracted primary molar

**Fig. 8:**
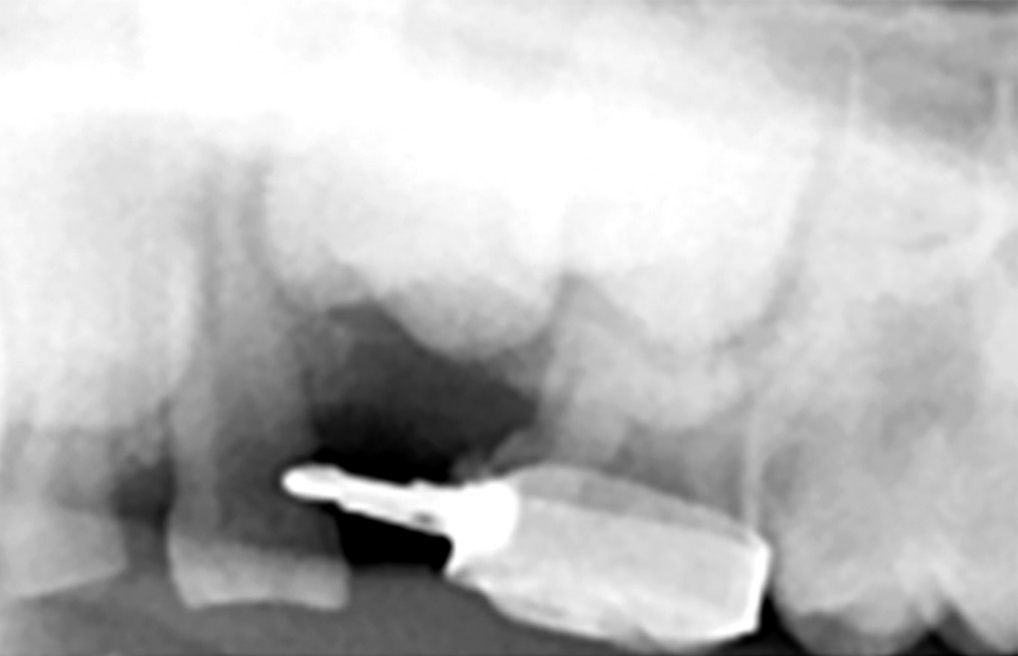
Band and loop space maintainer placed after extraction of submerged primary molar

On clinical examination, it was found that the left first primary molar was badly submerged (Fig. 4). An OPG was advised. It showed that the primary first molar was ankylosed (Fig. 5). So, the tooth was removed under general anesthesia followed by the placement of a prefabricated band and loop space maintainer (Figs 6 to 8).

## DISCUSSION

‘Bilateral submerged deciduous teeth’ is a rare condition.^6^ They have the potential to cause malocclusion because of over retention and delayed resorption of their roots thus preventing eruption of permanent teeth. The submerged teeth undergone a variable degree of root resorption and then become ankylosed to the bone.^7,8^ Once the adjacent permanent teeth get erupted, the ankylosed tooth appears submerged below the level of occlusion. This illusion is created due to continued growth of alveolar process in relation to adjacent permanent teeth; so that the relative level of occlusion gets changed. The cause of ankylosis is not known although in some cases trauma, infection, disturbed local metabolism or a genetic influence has been considered as an important etiologic factor. These influences have been discussed by Henderson who has also emphasized that a patient having one or two ankylosed teeth is very likely to have other teeth ankylosed over a period of time.^9^ This condition is usually treated by surgical removal of the ankylosed teeth so as to prevent the development of maloc-clusion, local periodontal disturbances, or dental caries.^5^
